# Left Atrial structure and function in hypertrophic cardiomyopathy sarcomere mutation carriers with and without left ventricular hypertrophy

**DOI:** 10.1186/s12968-017-0420-0

**Published:** 2017-12-28

**Authors:** Hoshang Farhad, Sara B. Seidelmann, Davis Vigneault, Siddique A. Abbasi, Eunice Yang, Sharlene M. Day, Steven D. Colan, Mark W. Russell, Jeffrey Towbin, Mark V. Sherrid, Charles E. Canter, Ling Shi, Michael Jerosch-Herold, David A. Bluemke, Carolyn Ho, Tomas G. Neilan

**Affiliations:** 1 Non-Invasive Cardiovascular Imaging Program and the Cardiovascular Division, Department of Medicine, Brigham and Women’s Hospital, Harvard Medical School, 75 Francis St, Boston, MA 02115 USA; 2 Radiology and Imaging Sciences, National Institutes of Health, Bethesda, MD USA; 3 Department of Cardiology, Johns Hopkins School of Medicine, Baltimore, MD USA; 4 Departments of Internal Medicine and Pediatrics, University of Michigan, Ann Arbor, MI USA; 5 Department of Cardiology, Boston Children’s Hospital, Boston, MA USA; 6 The Heart Institute and Pediatric Cardiology, Cincinnati Children’s Hospital Medical Center, Cincinnati, OH USA; 7 New York University Langone Medical Center, New York, NY USA; 8 Department of Pediatrics, Washington University School of Medicine, St. Louis, MO USA; 9 New England Research Institutes, Watertown, MA USA; 10 Department of Radiology, Brigham and Women’s Hospital, Harvard Medical School, Boston, MA USA; 11 Cardiac MR PET CT Program, Division of Cardiology and Department of Radiology, Massachusetts General Hospital, Boston, USA; 12 Division of Cardiology, Department of Medicine, Cardiac MR PET CT Program, Department of Radiology, Massachusetts General Hospital, 165 Cambridge St, Boston, MA 02114 USA

**Keywords:** Cardiovascular magnetic resonance (CMR), Hypertrophic cardiomyopathy, Left Atrial function

## Abstract

**Background:**

Impaired left atrial (LA) function is an early marker of cardiac dysfunction and predictor of adverse cardiac events. Herein, we assess LA structure and function in hypertrophy in hypertrophic cardiomyopathy (HCM) sarcomere mutation carriers with and without left ventricular hypertrophy (LVH).

**Method:**

Seventy-three participants of the HCMNet study who underwent cardiovascular magnetic resonance (CMR) imaging were studied, including mutation carriers with overt HCM (*n* = 34), preclinical mutation carriers without HCM (*n* = 24) and healthy, familial controls (*n* = 15).

**Results:**

LA volumes were similar between preclinical, control and overt HCM cohorts after covariate adjustment. However, there was evidence of impaired LA function with decreased LA total emptying function in both preclinical (64 ± 8%) and overt HCM (59 ± 10%), compared with controls (70 ± 7%; *p* = 0.002 and *p* = 0.005, respectively). LA passive emptying function was also decreased in overt HCM (35 ± 11%) compared with controls (47 ± 10%; *p* = 0.006). Both LAtotal emptying function and LA passive emptying function were inversely correlated with the extent of late gadolinium enhancement (LGE; p = 0.005 and *p* < 0.05, respectively), LV mass (*p* = 0.02 and *p* < 0.001) and interventricular septal thickness (*p* < 0.001 for both) and serum NT-proBNP levels (*p* < 0.001 for both).

**Conclusion:**

LA dysfunction is detectable by CMR in preclinical HCM mutation carriers despite non-distinguishable LV wall thickness and LA volume. LA function appears most impaired in subjects with overt HCM and a greater extent of LV fibrosis.

**Electronic supplementary material:**

The online version of this article (10.1186/s12968-017-0420-0) contains supplementary material, which is available to authorized users.

## Background

Hypertrophic cardiomyopathy (HCM) is caused by mutations in genes encoding sarcomere proteins [1]. Impaired left ventricular (LV) relaxation, altered myocardial energetics, and increased myocardial extracellular volume (ECV) have been demonstrated in HCM subjects without clinical disease, indicating that sarcomere mutations cause cardiac abnormalities prior to the establishment of LV hypertrophy (LVH) [2–5]. While the clinical diagnosis of overt HCM is defined by exceeding a threshold of LV wall thickness, typically greater than 1.3 to 1.5 cm, genetic testing allows for the early identification of at-risk family members when LV wall thickness is still within the normal range (LVH−) [6]. This unique scenario allows for the study of early-stage disease in sarcomere mutation or genotype-positive/LVH-negative (G+/LVH−) individuals, herein referred to as “preclinical HCM”.

Historically, LV morphology and function have been extensively studied in HCM; however, evaluation of the left atrium (LA) has been largely underutilized [7, 8]. An increase in LA volume (LAV) has been established as a marker of adverse cardiac events among broad groups of patients with cardiovascular disease [9–13]. Recently, data have shown that metrics of LA function can provide prognostic information beyond measurement of LA volume. For example, early detection of LA dysfunction may provide additional insight into the pathophysiology and aid in the clinical management of common cardiovascular diseases such as atrial fibrillation, myocardial ischemia, heart failure and cardiomyopathy [9, 10, 12].

LA function can be comprehensively assessed by measuring the three components that contribute to LV filling: reservoir, conduit, and pump phases. The reservoir phase starts at ventricular systole and encompasses LV isovolumetric contraction, ejection, and isovolumetric relaxation. During the reservoir phase, blood is received from the pulmonary veins and the LA fills, growing in volume as the mitral valve remains closed. Next, the LA acts as a conduit as the mitral valve opens and passive emptying occurs. LAV decreases during early ventricular diastole, driven by a suction effect on the LA as the LV relaxes and expands. Lastly, the atrial pump phase occurs during late ventricular diastole as the atrial muscle contracts and executes an active pump function, concluding ventricular filling. Technically, the LA reservoir, conduit, and active pump functions can be calculated from LAV at its maximum (end-systole, just prior to mitral valve opening), minimum (end-diastole, at mitral valve closure), and immediately before atrial contraction (before the electrocardiographic P-wave). From these three LAVs, total, passive, and active emptying functions can be calculated (LATEmF, LAPEmF and LAAEmF).

In this study, we hypothesized that LA function is abnormal in preclinical HCM mutation carriers, declines further in those with overt HCM, and correlates with other metrics of disease severity. To test these hypotheses, we examined the relationship between LA function, clinical parameters and established biomarkers for heart failure in a well-characterized genotyped cohort with preclinical and overt HCM and mutation-negative healthy family members.

## Methods

### Study population

HCMNet is a collaborative network comprised of 11 HCM specialty centers in the United States (Additional file 1: Table S1) [14]. This cross-sectional observational study was performed from 2009 to 2011 of sarcomere mutation carriers with clinically overt HCM (G+/LVH+), mutation carriers without LVH (G+/LVH-), and healthy relatives who do not carry the family’s mutation (G−/LVH- controls). LVH was based on echocardiographic core laboratory measurements and defined as a maximal LV wall thickness ≥ 12 mm in adults or a z-score ≥ 3 in participants <18 years of age. Genotype was confirmed in CLIA-approved laboratories either prior to or in conjunction with study enrollment. Variant interpretation was based on standard criteria accounting for segregation, conservation, and absence from appropriate control populations [15]. Sarcomere mutations were required to be classified as pathogenic or likely pathogenic by the testing laboratory at enrollment. Pathogenicity was reassessed at the time of data analysis and only those that fulfilled current criteria as pathogenic or likely pathogenic were included.

The 11 HCMNet centers were responsible for recruiting and enrolling participants and carrying out study procedures with standardized protocols. Institutional review boards at each participating site approved the study protocol and all participants provided written informed consent or parental consent/youth assent. As part of the HCMNet protocol, subjects underwent transthoracic echocardiography, exercise testing and assessment of serum biomarkers. CMR was performed for study participants when there were no contraindications (e.g., implantable cardiac device). Of 111 CMR studies performed in HCMNet, approximately 20% (*N* = 24) could not be scored due to the lack of diagnostic views in both 2- and 4-chamber views necessary to accurately assess LA volumes. Fourteen children under 15 years of age were not included due to currently undefined reference values and z-scores for LA function in children and the lack of normal controls to provide such values in this study cohort (*n* = 4). Therefore, a total of 73 studies were scored for LA volumes and function in this study.

### CMR protocol

All images were acquired with electrocardiographic gating, breath-holding, and with the patient in a supine position as previously described [3]. Each participant was imaged on a 1.5 T or 3 T CMR system depending on site technical availability. The standard CMR protocol consisted of balanced cine steady-state free precession imaging for cardiac function and mass (repetition time, 2.4 ms; echo time, 1.2 ms; spatial resolution, 1.4 × 1.8 × 8 mm, temporal resolution ≤40 msec) [16]. For late gadolinium enhancement (LGE) imaging, a segmented inversion-recovery pulse sequence was used starting 10–15 min after a single bolus dose of 0.15-mmol/kg of gadolinium DTPA (Magnevist®, Bayer HealthCare, Whippany, New Jersey, USA). Cine imaging and LV LGE imaging were obtained in 8 to 14 matching short-axis (8 mm thick with 0 mm spacing) as well as 2, 3 and 4 chamber long-axis planes [9]. LV mass was determined by tracing successive short axis images (excluding papillary muscles from the volume) and multiplying the myocardial muscle volume by 1.05 g/cm, indexed to body surface area (BSA) [17] (CIM software package version 6.2; Auckland MR Imaging Research Group, University of Auckland, Auckland, New Zealand) [18]. The thickness of the LV wall was measured throughout the myocardium with the largest measurement in each of four segments (anterior and posterior septal, lateral and inferior) defined as the maximal wall thickness.

The extent of LGE was quantified by a semi-automatic detection method using a previously validated research tool (QMassMR, version 7.4; Medis, Leiden, the Netherlands). The mass of LV LGE was measured in grams and was expressed as a percentage of the total LV myocardial mass. The core laboratory (Radiology and Imaging Sciences Department, National Institutes of Health Clinical Center) performed all CMR analyses by staff blinded to patient genotype.

### Left atrium analysis

A commercial software package (CVI 42, Circle Cardiovascular Imaging, Calgary, Canada) was used to analyze LA emptying function. LAV were measured at (1) the beginning of LV diastole (defined as the frame immediately prior to opening of the mitral leaflets, LAV_max_), (2) the end of passive LV filling (defined as the frame immediately prior to LA contraction, LAV_bac_), and (3) the end of LA contraction (LAV_min_). The inferior LA border was defined as the plane of the mitral annulus based on prior convention [9, 19]. To calculate LAV, we measured LA length (from the midpoint of the mitral annulus plane) and border (excluding the LA appendage and pulmonary veins) in the two- and four-chamber views. We then applied the biplane area-length method: LAV = [(8/3π) x (4-chamber area) x (2-chamber area)]/atrial length [19, 20]. As previously described [9, 10], LAPEmF was calculated as (LAV_max_ - LAV_bac_)/LAV_max_ × 100; LAAEmF as (LAV_bac_ – LAV_min_)/LAV_bac_ × 100; and LATEmF as (LAV_max_ - LAV_min_)/ LAV_max_ × 100. Measurements were made by investigators (HF and DV) blinded to patient genotype and clinical history.

### Transthoracic Echocardiography

Standard two-dimensional images, spectral and color flow Doppler, and tissue Doppler interrogation were obtained. Measurements were determined from the mean of 3 cardiac cycles in accordance with guidelines of the American Society of Echocardiography [21]. Early myocardial tissue Doppler relaxation velocities (E’) were measured at the lateral, septal, anterior, and inferior aspects of the mitral annulus. Global E’ velocity was determined by averaging these four values. All echocardiographic studies were analyzed offline by the echocardiographic core laboratory (The Johns Hopkins Echocardiography Research Laboratory, Baltimore, Maryland, USA), blinded to genotype status.

### Biomarkers

Blood samples were obtained at the time of cardiac imaging, processed within 60 min of phlebotomy, and stored at −80 degrees Celsius prior to analysis. Assays for NT-proBNP (Roche, Indianapolis, IN), galectin-3, soluble ST, and supersensitive cardiac troponin I (Singulex, Atlanta, Georgia, USA) were performed by the biomarker core laboratory blinded to clinical and genetic status.

### Statistical analysis

Continuous data are presented as mean ± standard deviation (SD), and categorical variables are summarized with frequencies and percentages. Generalized linear regression was used to compare baseline characteristics and LA volume and function across the three groups (G+/LVH+, G+/LVH-, G−/LVH- controls) adjusting for age, gender and body surface area (BSA). The clustered robust standard errors option in STATA was used in the linear regression model to account for observations clustering within family. Post-hoc Bonferroni-corrected *P* values <0.017 (0.05/3 groups for comparison) were considered to indicate statistical significance for multiple comparisons across the three groups. Intra-observer variability was not performed but inter-observer variability was tested by a second analysis. The coefficient of variation for each of the measures of LAV was calculated as the SD divided by the mean. Multivariate linear regression was used to examine the relationship between LAV and function measures and other echocardiographic, CMR and biomarker parameters by using LAV and function measures as the outcome (Y) and other echo, CMR and biomarker parameters as the predictor (X), adjusting for age, gender, BSA and familial relationships. Linear regression models were also used to assess the association between measures of LA function and controls, preclinicals and overt HCM subjects further stratified by extent of LGE. A cut-off value of 4 % was used corresponding to the median value of LGE. Stata 14.1 was used for statistical analysis (StataCorp LP, College Station, Texas, USA).

## Results

Basic demographics of the study population are presented in Table 1. Mean age, gender, blood pressure and BMI did not differ between the control (*N* = 15) and preclinical groups (*N* = 24) but overt HCM subjects (*N* = 34) were 6 to 9 years older (*p* < 0.001). LV wall thickness did not differ between the control and the preclinical groups. LV wall thickness was greater in the overt HCM group as compared to preclinical and control cohorts (p < 0.001 for both). LVEF and LV mass was similar between controls and preclinical subjects but was higher in subjects with overt HCM (<0.01 for both). LV end diastolic volume and maximal LV outflow tract velocity did not differ between the 3 groups. Serum NT-proBNP levels were significantly higher in the overt HCM group compared with controls and preclinical HCM (*p* < 0.001 for both). LGE was only observed in the overt HCM group but not in the control and preclinical cohorts.

**Table 1:** Baseline characteristics in sarcomere mutation carriers with and without left ventricular hypertrophy compared to healthy mutation-negative family member controls

	G+/LVH+ HCM (*N* = 34)	G+/LVH- Preclinical (*N* = 24)	G−/LVH- Control (*N* = 15)	*P*-value* overall	*P*-value* Preclinical vs Control	*P*-value* HCM vs Preclinical	*P*-value* HCM vs Control
Age, years	33 ± 12	27 ± 10	24 ± 6	<0.001	0.1	<0.001	<0.001
Male, *n* (%)	25 (74%)	12 (50%)	7 (47%)	0.003	0.8	0.008	0.007
G+ mutation, *n* (%)							
MYH7	6 (18%)	11 (46%)	0				
MYBPC3	21 (62%)	10 (42%)	0				
TNNT2	5 (14%)	1 (4%)	0				
TNNI3	1 (3%)	1 (4%)	0				
MYL2	1 (3%)	0 (0%)	0				
MYL3	0 (0%)	0 (0%)	0				
ACTC	0 (0%)	1 (4%)	0				
BMI,kg/m2	28.0 ± 4.3	24.0 ± 3.8	27.0 ± 4.5	0.4	0.6	0.2	0.4
SBP, mmHg	120 ± 11	115 ± 13	122 ± 14	0.7	0.5	1.0	0.7
DBP, mmHg	70 ± 7	70 ± 8	71 ± 9	0.6	0.8	0.4	0.7
IVS, mm	16.0 ± 5.3	9.0 ± 1.2	8.9 ± 1.2	<0.001	0.5	<0.001	<0.001
PW, mm	10.0 ± 2.2	7.9 ± 1.2	7.8 ± 1.6	<0.001	0.3	<0.001	<0.001
Maximal LVWT, mm	16.0 ± 5.1	10.0 ± 2.1	9.7 ± 1.5	<0.001	0.7	<0.001	<0.001
LVEF, %	66 ± 9.4	63.0 ± 6.3	60.0 ± 4.4	0.003	0.8	0.008	<0.001
Max LVOT, m/s	1.3 ± 0.2	1.3 ± 0.2	1.6 ± 0.5	0.036	0.7	0.052	0.2
LV mass, g	189 ± 53	121 ± 29	136 ± 34	<0.001	0.1	<0.001	<0.001
LV massi, g/m^2^	93 ± 24	66 ± 15	72 ± 15	<0.001	0.2	<0.001	0.012
LVEDVi, ml/ m^2^	45 ± 14	47 ± 9.3	51 ± 14	0.4	0.5	0.6	0.4
Septal e’ (cm/s)	9.8 ± 2.9	12 ± 2.0	14 ± 2.0	<0.001	0.003	0.047	<0.001
Septal e’/a’ ratio	1.2 ± 0.4	1.7 ± 0.5	1.9 ± 0.6	0.001	0.026	0.3	<0.001
Lateral e’ (cm/s)	14 ± 4.2	16 ± 2.4	16 ± 3.0	0.4	0.6	0.5	0.6
Lateral e’/a’ ratio	1.7 ± 0.8	2.2 ± 1.0	2.5 ± 1.0	0.15	0.6	0.7	0.2
Global E’, cm/s	12 ± 3.4	15 ± 1.9	16 ± 2.3	<0.001	0.1	<0.001	<0.001
Log-NT-proBNP, pg/mL	286 ± 464	51 ± 43	30 ± 28	<0.001	0.5	<0.001	<0.001
LGE, *n*, % present	17 (50%)	0 (0%)	0 (0%)	–	–	–	–

LVH was based on echocardiographic core laboratory measurements, defined as a maximal LV wall thickness (LVWT) ≥12 mm in adults or a z-score ≥ 3 in participants <18 years of age. *LV* left ventricular, *SBP* systolic blood pressure, *DBP* diastolic blood pressure, *BSA* body surface area, *IVS* interventricular septum thickness measured by echocardiography, *PW* posterior wall thickness measured by echocardiography, *Max LVWT* maximal left ventricular wall thickness, measured by MRI, defined as the largest measurement in each of four segments (anterior and posterior septal, lateral and inferior), *LVEF* left ventricular ejection fraction, *Max LVOT* maximal left ventricular outflow tract velocity, *LVEDVi* Left ventricular End Diastolic Volume indexed to BSA, *E’* tissue Doppler early diastolic myocardial relaxation velocity, *LGE* late gadolinium enhancement. Values represent mean and SD unless otherwise indicated

*Adjusted for age, gender, BSA and family relationships; *P* values ≤0.017 were considered to indicate statistical significance, applying Bonferroni correction for multiple comparisons across the three groups

**Fig. 1:**
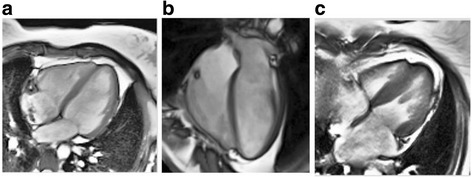
Representative CMR images from a control case (**a**), pre-clinical case (**b**) and an overt HCM case (**c**)

**Fig. 2:**
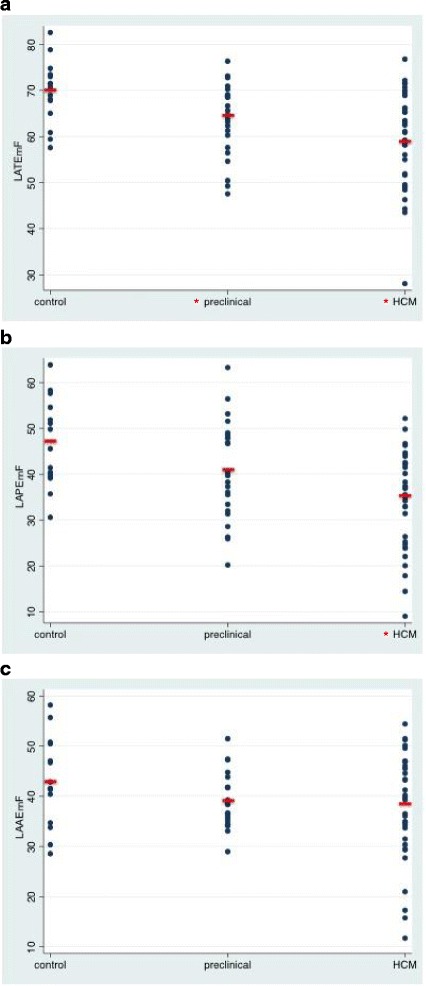
Scatterplots depicting the measured (**a**) Total Left Atrial Function (LATEmF) (**b**) Left Atrial Passive Function (LAPEmF) and (**c**) Active (LAAEmF) Emptying Function in Normal Controls (G-LVH-), subjects with sarcomere mutations without Left Ventricular Hypertrophy (preclinical, G + LVH-), and sarcomere mutation HCM subjects with overt LV hypertrophy (G + LVH+). Mean values are depicted by horizontal lines. *P values ≤0.017 versus control after adjusting for age, gender, BSA and family relationships

**Fig. 3:**
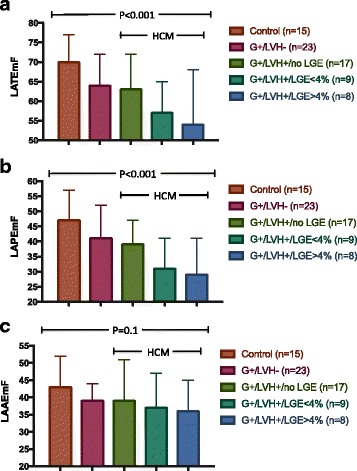
Bar graphs depicting the measured (**a**) Total (LATEmF) and (**b**) Passive (LAPEmF) and (**c**) Active (LAAEmF) Emptying Function in Normal Controls (G-LVH-), subjects with sarcomere mutations without LVH (preclinical, G + LVH-), and sarcomere mutation HCM subjects with overt LVH (G + LVH+) further stratified by having no LGE, ≤4% LGE and >4% LGE by CMR assessment. Adjusted for age, sex and BSA

The analysis of LA size was performed by two reviewers. We found a mean bias of 1.6 ± 8.5 mls for the LAVmin, of 2.9 ± 6.1 mls for the LAVmax and of 1.6 ± 5.8 mls for the LAVbac between reviewers, with a coefficient of variation ranging from 0.11 to 0.19. Representative images are provided in Fig. 1. LAV indexed to BSA were numerically larger in overt HCM subjects, but did not differ significantly between the control, preclinical HCM and overt HCM subjects after adjustment for age, gender, BSA and family relationships (Table 2). In contrast, measures of LA function differed between the 3 groups, as summarized in Table 2. The most consistent relationship was observed for LATEmF, which was lower in both the preclinical HCM group (64 ± 8%) and the overt HCM group (59 ± 10%) as compared to normal controls (70 ± 7%; *p* = 0.002 and *p* = 0.005, respectively; Table and Fig. 1a). LATEmF did not differ significantly comparing overt with preclinical HCM (*p* = 0.2, Table 2). Additionally, LAPEmF was lower in the overt HCM group (35 ± 11%) as compared to controls (47 ± 10%, *p* = 0.006; Table 2 and Fig. 2b). In analyses further stratified by the extent of LGE, overall, LATEmF and LAPEmF showed an incremental decline in control, preclinical and overt HCM groups; smallest in individuals with more LGE enhancement (*p* < 0.001 for trend for both; Fig. 3a and b). This pattern was not seen for LAAEmF (*p* = 0.1, Fig. 3c). Furthermore, while defining cut-offs to differentiate control from overt HCM, and control from preclinical patients is beyond the scope of this study, measurement of the area under the curve (AUC) for LA function yield the following results: the overall AUC for LATEmF is 0.28 for preclinical and 0.23 for HCM. Using a cutoff of 58% for LATEmF, the AUC is 0.60 for preclinical and 0.60 for HCM. A LATEmF cutoff of 28% is 100% sensitive and 0% specific for HCM, correctly classifying 63% of subjects. A LAREmF cutoff of 47.5% is 100% sensitive and 0% specific for preclinical status, correctly classifying 56% of subjects.

**Table 2:** CMR Imaging assessment of left atrial volumes and function in sarcomere gene mutation carriers with and without left ventricular hypertrophy compared to healthy family members

	G+/LVH+ Overt HCM (*N* = 34)	G+/LVH- Preclinical (*N* = 24)	G−/LVH- Controls (*N* = 15)	*P*-value overall*	*P*-value* Preclinical vs Control	*P*-value* HCM vs Preclinical	*P*-value* HCM vs Control
LAV Max/BSA, ml/m^2^	46 ± 18	32 ± 12	33 ± 9	0.031	0.83	0.050	0.13
LAV BAC/BSA, ml/m^2^	31 ± 16	20 ± 9	18 ± 6	**0.015**	0.56	0.050	0.10
LAV Min/BSA, ml/m^2^	20 ± 12	12 ± 5	10 ± 4	0.019	0.20	0.047	0.12
LAPEmF, %	35 ± 11	41 ± 11	47 ± 9	**0.002**	0.077	0.23	**0.006**
LAAEmF, %	38 ± 11	39 ± 5	43 ± 9	0.033	0.060	0.46	0.074
LATEmF, %	59 ± 10	64 ± 8	70 ± 7	**<0.001**	**0.002**	0.20	**0.005**

LVH was based on echocardiographic core laboratory measurements, defined as a maximal LV wall thickness (LVWT) ≥12 mm in adults or a z-score ≥ 3 in participants <18 years of age. *LAV* left atrial volume, *BAC* before atrial contraction, *BSA* body surface area, *LATEmF* Total left atrial function, *LAPEmF* left atrial passive function, *LAAEmF* left atrial active function. Values represent mean and SD unless otherwise indicated

*Adjusted for age, gender, BSA and family relationships; *P* values ≤0.017 were considered to indicate statistical significance, applying Bonferroni correction for multiple comparisons across the three groups

Multivariate regression models were constructed to assess the relationship between LA function and measures of cardiac structure, function and serum biomarkers (Table 3). All 3 measures of LAV were positively correlated with LV mass, the extent of LGE, and serum NT-proBNP after adjustment for age, gender, BSA and family relationship (*p* ≤ 0.01 for all, Table 3). In analyzing LA function, both LATEmF and LAPEmF were inversely correlated with these metrics (*p* < 0.05 for all), as well as with interventricular septal thickness (*p* ≤ 0.001 for both). LV end-diastolic volumes and LV end-systolic volumes were correlated with LAVmax but not any measures of LA function. Tissue Doppler early diastolic myocardial relaxation velocity (global E’) was positively associated with LAPEmF (*p* = 0.05) and negatively associated with LAVmin, LAVbac (*p* < 0.05 for both). No measure of LAV or function correlated with LVEF or serum levels of high sensitivity troponin I, matrix metalloproteinase-1 (MMP1), or matrix metalloproteinase inhibitor-1 (TIMP1).

**Table 3:** Regression analysis of left atrial function and measures of cardiac structure, function and serum biomarkers in the HCMnet cohort (*N* = 73)

	LAPEmF	LAAEmF	LATEmF	LAVmin*	LAVbac*	LAVmax*
IVS	−0.88 (.18) **<0.001**	−0.28 (.19) 0.1	−0.70 (.20) **0.001**	0.57 (.28) **0.046**	0.85 (.36) **0.023**	0.72 (.43) 0.097
LVEF	−0.088 (.18) 0.6	0.23 (.17) 0.2	−0.10 (.20) 0.6	−0.24 (.24) 0.3	−0.25 (.30) 0.4	−0.30 (.32) 0.4
LAVmin*	−0.007 (0.001) **<0.001**	−0.005 (0.0007) **<0.001**	−0.008 (0.0008) **<0.001**	–	–	–
LAVbac*	−0.005 (0.0007) **<0.001**	−0.002 (0.0007) **0.018**	−0.005 (0.0008) **<0.001**	–	–	–
LAVmax*	−0.002 (.0008) **0.004**	−0.002 (.0009) 0.058	−0.003 (.0009) **0.003**	–	–	–
LVEDV	−0.004 (.040) 0.9	−0.034 (.043) 0.4	−0.023 (.039) 0.6	0.075 (.044) 0.092	0.11 (.059) 0.073	0.17 (.08) **0.027**
LVESV	0.053 (.086) 0.5	−0.087 (.083) 0.3	−0.028 (.086) 0.7	0.14 (.10) 0.2	0.19 (.13) 0.1	0.32 (.16) **0.046**
LV mass	−0.099 (.022) **<0.001**	0.002 (.026) 1.0	−0.060 (.025) **0.022**	0.083 (.024) **0.001**	0.13 (.03) **<0.001**	0.13 (.04) **0.001**
Extent LGE	−0.43 (.20) **0.046**	−0.55 (.24) **0.034**	−0.74 (.22) **0.005**	1.23 (.26) **<0.001**	1.35 (.33) **<0.001**	1.32 (.37) **0.002**
Septal e’ (cm/s)	0.01 (.005) 0.067	−0.001 (.004) 0.8	0.005 (.004) 0.3	−0.77 (.40) 0.06	−1.20 (.55) **0.035**	−1.09 (.73) 0.14
Septal e’/a’ ratio	0.04 (0.02) 0.13	−0.02 (.02) 0.3	0.008 (.01) 0.7	−0.31 (2.2) 0.9	−1.77 (2.9) 0.55	−1.8 (3.6) 0.6
Lateral e’ (cm/s)	0.004 (.005) 0.4	−0.001 (.003) 0.6	0.002 (.004) 0.6	−0.49 (.37) 0.19	−0.75 (.50) 0.14	−0.54 (.60) 0.4
Lateral e’/a’ ratio	0.004 (.02) 0.8	−0.02 (.008) **0.032**	−0.008 (.01) 0.5	0.73 (1.2) 0.5	0.53 (1.9) 0.8	1.25 (2.4) 0.6
Global E’	0.97 (.48) **0.050**	0.021 (.41) 1.0	0.64 (.47) 0.2	−1.02 (.48) **0.037**	−1.50 (.54) **0.021**	−1.39 (.77) 0.079
Log-NT-proBNP	−0.040 (.006) **<0.001**	−0.014 (.01) 0.16	−0.035 (.008) **<0.001**	3.55 (1.1) **0.002**	4.53 (1.2) **<0.001**	3.99 (1.6) **0.013**
Troponin I	2.1 (56) 1.0	−73 (48) 0.2	−78 (63) 0.2	146 (73) 0.069	130 (74) 0.1	86 (99) 0.4
MMP1	0.21 (.002) 0.5	0.10 (.21) 0.5	0.19 (.14) 0.2	−0.18 (0.20) 0.4	−0.27 (0.30) 0.4	−0.16 (0.44) 0.7
TIMP1	−0.002 (.005) 0.7	0.004 (.003) 0.2	0.004 (.004) 0.3	−0.005 (.005) 0.3	0.001 (.006) 0.3	0.008 (.007) 0.2
GAL3	0.24 (.46) 0.6	0.056 (.28) **0.049**	0.53 (.36) 0.1	−0.50 (.30) 0.1	−0.60 (.43) 0.2	−0.83 (.55) 0.1
sST2	**−0.21 (.10) 0.043**	0.072 (.096) 0.5	−0.077 (.095) 0.4	−0.027 (.076) 0.7	−0.051 (.10) 0.6	−0.22 (.14) 0.1

Reported as beta coefficient (SE) *p*-value in the regression model with left atrial function as the outcome (Y) and other echo, CMR and biomarker measures as predictor (X), adjusted for age, gender, BSA, and family relationship

*IVS* interventricular septum thickness, *LVEF* left ventricular ejection fraction, *LVEDV* Left ventricular End Diastolic Volume, *LVESV* Left ventricular End Systolic Volume, *LV* left ventricular, *LGE* late gadolinium enhancement, *E’* tissue Doppler early diastolic myocardial relaxation velocity, N-terminal pro-B-type natriuretic peptide (NT-proBNP); high sensitive troponin I (troponin I); matrix metalloproteinase-1 (MMP1); matrix metalloproteinase inhibitor-1 (TIMP1); galectin-3 (GAL3). *indexed to BSA

## Discussion

In this study, we leveraged a robustly characterized study population to investigate whether CMR-derived measures of LA size and function differed between healthy controls and sarcomere mutation carriers with preclinical and overt HCM. We identified the following major findings: 1. LAV did not significantly differ between mutation carriers with preclinical or overt disease or controls; 2. LA dysfunction, reflected by reduced LATEmF, is detectable in sarcomere mutation carriers with both preclinical and overt HCM compared with healthy controls; 3. Patients with overt HCM, particularly those with the greatest amount of LGE, demonstrated the most marked reduction of LA function, with decreased LATEmF and LAPEmF; 4. Impaired LA function and increased LV volumes were associated with higher serum NT-proBNP levels and a greater extent of LGE.

These data provide additional insights regarding early phenotypic manifestations in preclinical HCM sarcomere mutation carriers. Our work suggests that changes in LA function precede changes in LA size in the pathogenesis of HCM sarcomere mutations. In the preclinical cohort, all 3 measures of LA size were similar to those of control subjects. Yet when LA function was assessed, the LATEmF was lower in preclinical HCM, and reduced in overt HCM. Given our prior work demonstrating increased extracellular volume fraction, a quantitative measure of the extent of extracellular expansion and a marker of interstitial fibrosis, in preclinical HCM subjects [3], we speculate that the impaired LA function seen in this cohort reflects altered myocardial tissue properties. The reduction in total LA function was primarily reflected in the passive emptying component, while the active emptying function was less affected.

Another interesting finding of this study was that the overt HCM group had significantly lower LATEmF and LAPEmF compared to preclinical HCM and controls. Further, when the overt HCM group was stratified by the presence or absence and extent of LGE, most of this difference was due to overt HCM subjects with a greater extent of LGE. We speculate that the development of fibrotic changes in the ventricular myocardium may precipitate a decline in LA function, particularly LAPEmF. Although a primary atrial myopathy may also be present, these findings suggest that alterations in myocardial tissue composition may also drive the development of LA dysfunction in HCM.

While defining cut-offs to differentiate control from overt HCM, and control from preclinical patients is beyond the scope of this study (due to the limitations of sample size as well as the fact that we do not have data as to whether any of our preclinical progress to overt HCM), we provide AUC results. However, this study focuses instead on metrics of LA size and function to better characterize the pathophysiologic impact of sarcomere mutations and therefore help elucidate underlying disease biology.

Fully understanding the clinical implications of impaired LA function in either overt or preclinical HCM will require further longitudinal investigation. However, a reduction in LA function has been shown to have prognostic implications in broad populations [9, 10]. For example, among patients referred for a stress CMR, a reduction in LAPEmF was shown to be a significant predictor of adverse cardiac events [10]. Similarly, among patients with atrial fibrillation, a reduction in LAPEmF was shown to be a robust predictor for recurrence after pulmonary vein isolation [10]. In this study, LA total function was abnormal and passive function trended lower in the preclinical group. It remains to be studied whether reduced LA function will be associated with earlier progression to overt HCM, a greater susceptibility to atrial fibrillation, more prominent symptoms of heart failure, and/or poorer prognosis in these subjects. However, some prior data have shown the association between measures of LA size and function and adverse outcomes in HCM [7, 8]. For example, in a study of 242 HCM patients Debonnaire and colleagues showed than LAV and LA strain were predictive of new onset AF in patients with HCM [8].

The penetrance or clinical expression of a HCM gene mutation is age dependent and commonly occurs during young adulthood. When phenotypic conversion does occur, LV wall thickness can increase rapidly and the pathological manifestations of HCM may arise suddenly. In line with its natural history, subjects with overt HCM in this study were slightly older than preclinical and control groups. Much has yet to be understood of the preclinical stages of HCM. In subjects who are at risk, based on their family history and genetic makeup, it is currently unpredictable when and if the overt HCM will manifest. Developing non-invasive tools to potentially identify those subjects at greater risk for disease onset would be important for clinical management. These subjects could potentially be followed more closely with physician visits and echocardiographic assessment.

Our study has several limitations. We describe a small study group without follow up to evaluate clinical outcomes or longitudinal disease evolution. As an observational study, inferences about causality cannot be determined. Furthermore, we did not measure atrial LGE or measure the ECV to correlate with our finding of LA dysfunction in sarcomere mutation carriers. Lastly, 20% of the cohort did not have both 2 and 4-chamber views needed to quantify LA function. However, when we compared baseline characteristics between those subjects that underwent CMR and had quantifiable LA function versus those that were unable to be quantified, we found no significant differences between groups.

## Conclusion

In conclusion, in a well-characterized and genotyped cohort of subjects with preclinical HCM, overt HCM and familial controls, we found that LA dysfunction is detectable in preclinical HCM subjects before the presence of LA enlargement or LVH. Furthermore, LA dysfunction is more pronounced as LVH and ventricular fibrosis are manifest. Impaired LA function appears to have physiologic relevance, being associated with a greater extent of LGE and higher NT-proBNP levels. These data support the hypothesis that HCM is a progressive disorder in which at-risk sarcomere mutation carriers begin to have physiological manifestations before clinical criteria for overt disease are met.

## Additional file

Additional file 1: Table S1. HCMNet Participating Sites and Enrollment. (DOCX 12 kb)

## Abbreviations

(G−/LVH- controls): Healthy relatives who do not carry the family’s mutation (Genotype negative) and without LVH
(G+/LVH-): Mutation carriers (Genotype positive) without LVH
(G+/LVH+): Mutation carriers (Genotype positive) with LVH
AUC: Area under the curve
BMI: Body mass index
BSA: Body surface area
CMR: Cardiovascular magnetic resonance
HCM: Hypertrophic cardiomyopathy
LA: Left atrium/left atrial
LAAEmF: Active left atrial emptying function
LAPEmF: Passive left atrial emptying function
LATEmF: Total left atrial emptying function
LAV: Left atrial volume
LAV_bac_: LA volume before atrial systole
LAV_max_: Maximal left atrial volume
LAV_min_: Minimal left atrial volume
LGE: Late gadolinium enhancement
LV: Left ventricle/left ventricular
LVEF: Left ventricular ejection fraction
LVH: Left ventricular hypertrophy
SD: Standard deviation
