# Supplementary material for: Left Atrial structure and function in hypertrophic cardiomyopathy sarcomere mutation carriers with and without left ventricular hypertrophy
Source: J Cardiovasc Magn Reson. 2017 Dec 28;19:107. doi: 10.1186/s12968-017-0420-0 (PMC5745877; doi:10.1186/s12968-017-0420-0)
Supplement: Additional file 1: Table S1. — HCMNet Participating Sites and Enrollment. (DOCX 12 kb) [file 12968_2017_420_MOESM1_ESM.docx]

**Additional file 1:** **Table S1**.

| **Site** | **Site PI** | **Number of Subjects Enrolled** |
| --- | --- | --- |
| Brigham and Women’s Hospital | Carolyn Ho, MD | 62 |
| Boston Children's Hospital | Steve Colan, MD | 44 |
| Cleveland Clinic Foundation | Harry Lever, MD | 2 |
| Cincinnati Children’s Hospital Medical Center | Jeff Towbin, MD   John Lynn Jefferies, MD | 10 |
| University of Michigan | Sharlene Day, MD  Mark Russell, MD | 30 |
| University of Chicago | Elizabeth McNally, MD, PhD | 1 |
| St. Luke’s-Roosevelt Hospital Center | Mark Sherrid, MD  Bette Kim, MD | 10 |
| Johns Hopkins University | Anne Murphy, MD | 4 |
| Washington University School of Medicine | Charles Canter, MD | 10 |
| University of Colorado | Matthew Taylor, MD  Luisa Mestroni, MD | 4 |
| Stanford University | Euan Ashley, MRCP DPhil | 1 |
